# Nanoparticle- and Nanoporous-Membrane-Mediated Delivery of Therapeutics

**DOI:** 10.3390/pharmaceutics11060294

**Published:** 2019-06-21

**Authors:** Mostafa Mabrouk, Rajakumari Rajendran, Islam E. Soliman, Mohamed M. Ashour, Hanan H. Beherei, Khairy M. Tohamy, Sabu Thomas, Nandakumar Kalarikkal, Gangasalam Arthanareeswaran, Diganta B. Das

**Affiliations:** 1 Refractories, Ceramics and Building Materials Department, National Research Centre, 33 El Bohouth St (former EL Tahrirst)-Dokki, Giza 12622, Egypt; hananh.beherei@gmail.com; 2 International and Inter-University Centre for Nanoscience and Nanotechnology, Mahatma Gandhi University, Kottayam, Kerala 686560, India; rajimpharm@gmail.com (R.R.); sabuthomas@mgu.ac.in (S.T.); nkkalarikkal@mgu.ac.in (N.K.); 3 Biophysics Branch, Faculty of Science, Al-Azhar University, Cairo 11884, Egypt; islam_biophysics@yahoo.com (I.E.S.); already_a555@yahoo.com (K.M.T.); 4 Faculty of Engineering, Badr University, Cairo 11829, Egypt; mohamedashour739@gmail.com; 5 Department of Chemical Engineering, National Institute of Technology, Tiruchirappalli 620015, India; arthanareeg@nitt.edu; 6 Department of Chemical Engineering, Loughborough University, Loughborough LE113TU, UK

**Keywords:** pharmaceutical particulates, membranes, drug delivery systems, bio-imaging, bioactive molecules

## Abstract

Pharmaceutical particulates and membranes possess promising prospects for delivering drugs and bioactive molecules with the potential to improve drug delivery strategies like sustained and controlled release. For example, inorganic-based nanoparticles such as silica-, titanium-, zirconia-, calcium-, and carbon-based nanomaterials with dimensions smaller than 100 nm have been extensively developed for biomedical applications. Furthermore, inorganic nanoparticles possess magnetic, optical, and electrical properties, which make them suitable for various therapeutic applications including targeting, diagnosis, and drug delivery. Their properties may also be tuned by controlling different parameters, e.g., particle size, shape, surface functionalization, and interactions among them. In a similar fashion, membranes have several functions which are useful in sensing, sorting, imaging, separating, and releasing bioactive or drug molecules. Engineered membranes have been developed for their usage in controlled drug delivery devices. The latest advancement in the technology is therefore made possible to regulate the physico-chemical properties of the membrane pores, which enables the control of drug delivery. The current review aims to highlight the role of both pharmaceutical particulates and membranes over the last fifteen years based on their preparation method, size, shape, surface functionalization, and drug delivery potential.

## 1. Introduction

Nanotechnology has emerged as one of the most versatile and powerful technologies in the development of drug delivery techniques. Nanomaterials include particulates which have at least one dimension of 100 nm or less. Scale differences and modification to the material surfaces result in different physico-chemical properties of the materials which make them suitable for biomedical applications such as drug delivery, disease diagnosis, and therapy. Currently, there are many applications of nanotechnology in general and nanoparticle (NP)-based drug delivery specifically. Similarly, membrane technologies that focus on both nanoporous membrane preparation and applications have developed significantly. In this context, a membrane involves a porous system which is made up of either inorganic, organic, or a combination of both inorganic and organic materials. The utilization of membranes to convey medications/bioactives is opening up new treatment preferences of interest, e.g., enhancing the solubility of bioactives shielding an active ingredient from corruption, enhancing the bioavailability of medication, lowering lethal impacts, offering suitable structures for all courses of administration, permitting advancement and offering fitting structures for courses of drug administration, and permitting fast formulation improvement. These points are discussed further below. 

### 1.1. Why Nanoparticles?

There are many different types of NPs that have promising biomedical applications, e.g., polymeric NPs, polyethylene glycol (PEG)-ylation modified particles, micelles, liposomes, dendrimers, and nanosized inorganic materials [1]. Organic NPs vary in their activity in different biological systems including penetration depths in tissues and their toxicity and targeting efficiency [2]. Inorganic NPs regularly display novel physical properties as their size is close to nanometer scale measurements. For instance, the extraordinary physical and chemical properties of these NPs may prompt future applications in drug delivery and biomedical imaging. Plenty of sophisticated and different applications, including diagnosis and therapy and flow investigation, are related to configuration of the high surface-to-volume proportions of NPs as a potential system for these strategies. Structures such as core/shell NPs can show improved properties and increased usefulness due to their changed chemical distinction and nanostructured parts. This article therefore features an assortment of structures and properties that can be acknowledged in materials dependent on inorganic NPs and focuses on discussing major inquiries brought up in controlling these properties. 

**Figure 1:**
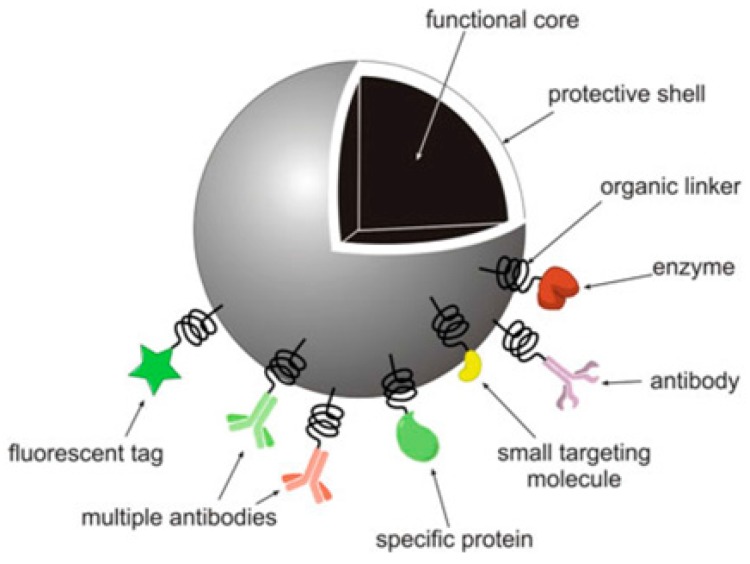
A model design of an inorganic nanoparticle (NP) functionalized with biomolecules for biomedical applications [6]. Reproduced with copyright permission from Springer Nature, 2010.

Typical characteristics of inorganic nanomaterials, such as ease of fabrication, modification and functionalization, simple preparation methods, resistance to microbial attacks, high stability and suitable size for cells (plasma membrane below 100 nm), a low toxicity profile, biocompatibility, and having a hydrophilic nature, make them suitable as drug carriers [3]. Moreover, these NPs can be biodegradable, non-toxic, non-immunogenic responsive, have a high loading capacity, and have the capacity for controlled drug release. These characteristics are desired for biomedical applications in addition to properties such as magnetism. However, in practice, it is not easy to fabricate ideal NPs which have all the desired properties [4,5] and some NPs are indeed not suitable for biomedical applications. To improve their biocompatibility, protective coatings which can be non-toxic, such as natural polymers (carbohydratesand peptides) or synthetic polymers (e.g., PEG, polyvinyl alcohol (PVA), and polyglycolic acid (PGA), etc.) can be used. Figure 1 shows a cut-out model of an inorganic NP functionalized with biomolecules for biomedical applications [6].

### 1.2. Why a Nanoporous Membrane?

Membranes have several biological functions which are useful in sensing, sorting, imaging, separating, and releasing bioactive/drug molecules. Engineered nanoporous and microporous membranes have been developed for their usage in drug delivery. The latest advancement in technology is therefore possible for use in regulating the physico-chemical properties of membrane pores, which make them attractive for controlling drug delivery rates. In addition, different types of materials are used for the fabrication of membranes and their properties and surface modification in order to improve the functions of the membranes, providing different invitro and in vivo applications of therapeutic delivery. In spite of the extensive work carried out for preparation, characterization, and biological evaluation of membranes, there are still a number of challenges which need to be overcome to develop biological membranes.

**Figure 2:**
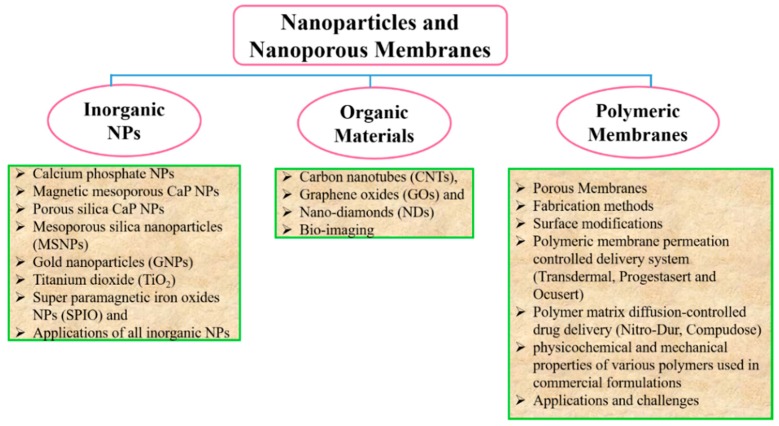
Structural overview of the article.

Membranes are used to control the rate of delivery of drugs to the body as well as drug permeation from the reservoir to attain the required rate of drug delivery. Therefore, drug delivery is controlled by both passive diffusion and biodegradation mechanisms. Membranes can carry one or more bioactive agents and have been developed into different classes of carriers. These different carriers can be carbon-based nanomaterials, polymeric membranes, and inorganic membranes, where the bulk properties of the membrane are governed by its building blocks, i.e., the NPs. Keeping this in mind, the current review aims to highlight the role of both pharmaceutical NPs and membranes during the last fifteen years based on their preparation method, size, shape, surface functionalization, and drug delivery potential. The following classification gives an overview structure of the article (Figure 2). 

## 2. Drug Delivery System

Since the emergence of controlled drug delivery systems (DDS) in early the 1970s, these systems have attracted increasing attention. DDS are aimed at delivering drugs using pre-defined doses and drug delivery rates. Moreover, the area of drug delivery is an expanding domain focused on targeting genes or drug formulations to a group of diseased cells or tissues. The objective of this technique is to carry an appropriate amount of drug to the target sites (such as diseased tissues and tumors, etc.) while limiting undesirable reactions of the drugs within healthy tissues [7]. The propensity of the carrier material to cure cancer has been influenced by various parameters that relate to the carrier material, e.g., the immune response to the carrier material and uncontrollable drug behavior [8]. The morphology of NPs also performs a significant role in drug loading and release and achieving the maximum cell viability and minimum cell morbidity [9]. In order to illustrate how NPs have been used as DDS, we provide a number of key examples below.

### 2.1. Calcium Phosphate NPs

Calcium phosphate (CaP) is a common NP used in biological systems and medical applications, especially within those related to diagnosis and treatment. In particular, CaP NPs are extensively used in imaging, bone/tooth repair, and DNA delivery in cell biology [10,11]. The widespread uses of CaP NPs are due to their presence as a natural component in the body, as CaP is well enhanced and easily absorbed in the circulatory system [12]. Hence, previous studies have shown that CaP NPs can be used as a drug carrier. Kester et al. [13] have assessed the ability of CaP NPs to be encapsulating hydrophobic antineoplastic chemotherapeutics. Furthermore, these NPs have shown their ability to encapsulate both fluorophores and chemotherapeutics. These NPs have a diameter of 20–30 nm and pH sensitivity, as well as little degree of disparity. In addition, these NPs have been observed to be steady in physiological solution for a time duration at a surrounding temperature of 37°C. Bastakoti et al. [14] have claimed that colloidal NPs smaller than 100 nm filled with fluorescent pigments and anticancer drugs have resulted in the successful enhancement of robust biocompatible nanocomposites carriers for simultaneous release of drug molecules and imaging agents.

Shinto et al. [15] have utilized hydroxyapatite porous ceramic blocks (a CaP-based material) NPs in order to formulate nanocarriers for sustained delivery of antibiotics. Firstly, the cylindrical cavities in the hydroxyapatite blocks were loaded with the antibiotic and they were further implanted in the bone defect sites. The results revealed that a higher concentration of the antibiotic was released after seven days of implant. Then, there was a gradual decrease in the concentration after 12 weeks. Overall, the release of antibiotics with an efficiency of 70% was obtained.

Zhao et al. [16] have investigated the drug delivery ability of anticancer drug docetaxel-loaded lipid-calcium phosphate hybrid NPs, where the NPs showed a high drug loading capacity and biocompatibility. The NPs used in this study had a diameter of 72 nm. Mukesh et al. [17] have also proposed the use of CaP NPs as a carrier for the anticancer drug methotrexate; the size of the NPs in their case was 262 nm, with an encapsulation efficiency of 58%.They showed a low release rate of methotrexate at physiological pH and the authors observed that over 90% release was obtained in 3 to 4 h at endosomal pH. Liang et al. [18] have examined the in vitro delivery of the anticancer drug doxorubicin (DOX) hydrochloride from CaP hybrid NPs with particle sizes smaller than 50nm. Heparin/CaCO_3_/CaP NPs were loaded with this anticancer drug. It was observed that the unloaded hybrid NPs showed high biocompatibility while the anticancer loaded NPs exhibited a strong cell inhibitory effect. These examples suggest a clear potential for CaP NP use in biomedical applications. CaP NPs have demonstrated successful delivery of drugs and bioactive molecules alone or in combination with polymers, owing to their biocompatibility. However, more research efforts should be focused in the future on investigating CaP NP degradation products in vivo and their effects on the vital organs.

#### 2.1.1. Magnetic Mesoporous CaP NPs

Magnetic mesoporous CaP NPs with high water solubility and a diameter of 41 nm have been fabricated by Rout et al. [19]. These CaP NPs were composed of platinum pharmacophorecis-diaquadiamine platinum, folic acid, and rhodamine isothiocyanate, in order to use them against human cervical carcinoma cells. It was observed that the targeting of these cancer cells and delivery of cisplatin could be achieved by utilizing magnetic CaP NPs, as confirmed by cell apoptosis that was followed by cell death. However, no information was reported about their clearance from the body on both bases of animal models and clinical trials.

#### 2.1.2. Porous Silica CaP NPs

El-Ghannam et al. [8] have used porous silica CaP NP as a carrier for 5-fluorouracil, which is considered very cytotoxic for 4T1 mammary tumor cells. The invitro study demonstrated that the NPs loaded with the anticancer drug possessed characteristics of burst release (the maximum release rate) in the first 24 h, and, following this, a sustained release, which was observed for the next 32 days. Meihuaet al. [20] have reported the preparation of mesoporous silica NPs coated with a CaP-hyaluronic acid hybrid. The core shell was further coated with another hyaluronic acid layer in order to target CD44 over-expressed cancer cells. The authors proved that the anticancer release in an acidic subcellular environment could be controlled using their NPs. These studies exhibited the superiority of core shell nano-systems over normal nanomaterials in the delivering of anticancer in a sustained manner.

### 2.2. Carbon-Based Nanomaterials

Other types of nanomaterials that may be utilized in therapeutic delivery, and especially for anticancer therapy, are carbon-based nanomaterials, including carbon nanotubes (CNTs), graphene oxides (GOs), and nano-diamonds (NDs), as discussed below.

#### 2.2.1. Carbon Nanotubes

When carbon atom nanostructures are arranged in a tube-like hollow cylindrical shape, they may be called carbon nanotubes (CNTs) [21]. These are divided into three categories: single-walled carbon nanotubes (SWNTs), double-walled carbon nanotubes (DWNTs), and multi-walled carbon nanotubes (MWNTs). Their diameters may range between several angstroms and tens of nanometers while their lengths may reach half a meter [22,23].

One of the biggest benefits of carbon nanomaterials in biomedical applications is their ability to be a drug carrier. Pastorin et al. [24] have reported that, as an anticancer drug, methotrexate linked via a covalent bond to carbon nanotubes with fluorescein isothiocyante (FITC) was more effectively internalized through folate receptors into cells in comparison to the free drug. CNTs have also been designed to be tumor targetable through functionalization to be used as drug carriers. Moreover, SWNTs have also been used as drug carriers in their water-soluble PEG-ylated form. They have demonstrated a loading capacity for the anticancer drug DOX [25]. MWNTs have been examined as a drug carrier, and in particular oxidized MWNTs have been PEG-ylated for anticancer drug specific delivery to the brain for the treatment of glioma. Other polymers have also been utilized as functionalizing agents for CNTs for drug delivery applications, as has been reported previously [26]. MWNTs have been functionalized with a polyethylenimine (PEI) composite, which has shown impressive biocompatibility. Furthermore, these composites were designed to be accompanied by FITC and prostate stem cell antigen (PSCA) monoclonal antibody (mAb). Finally, a CNT-PEI (FITC)-mAb composite has been obtained which showed impressive biocompatibility with a cancer-cell-targeted delivery system [27].

**Figure 3:**
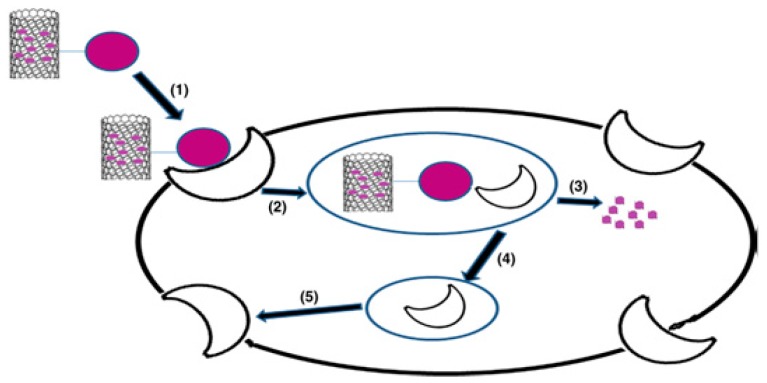
Receptor-mediated endocytosis of CNTs. (**1**) Association of ligand conjugated drug-loaded CNTs with receptor; (**2**) endosomal internalization of conjugates, (**3**) drug release, and (**4**,**5**) receptor regeneration [28]. Reproduced with copyright permission from Elsevier, 2016.

Cellular uptake of CNTs has been explained by Bhatt et al. [28]. They have suggested mechanisms for CNT cell-internalization via two pathways, namely, dependent and independent endocytosis pathways, where the endocytosis-independent pathway is divided into two types: receptor-mediated endocytosis and non-receptor-mediatedendocytosis (see Figure 3). CNTs with their available types either functionalized or not have demonstrated great success in the delivery of bioactive molecules, especially anticancer agents, and the targeting of tumor cells.This has encouraged scientists to explore their applicability within most common diseases other than cancer. Nevertheless, CNTs have not been explored widely, owing to their difficult and expensive preparation methods. 

#### 2.2.2. Graphene Oxide

Another carbon-based nanomaterial for use in biomedical applications is graphene oxide. Nano-graphene oxide complexes, especially metal nanocomposites (nanogold and nanosilver), have been found to be a good choice of material to treat cancer. Due to their greater drug entrapment capability and photothermal and synergizing effects, it is worth highlighting them for their targeted treatment of cancer based on their chemo-thermal behavior.

**Figure 4:**
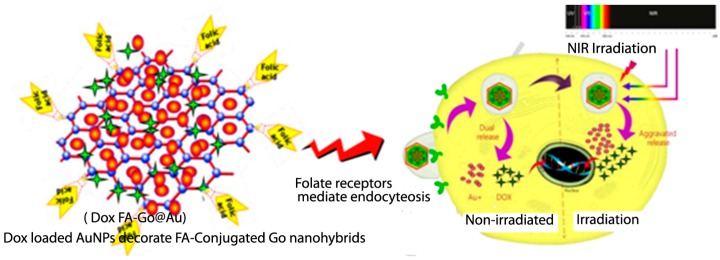
Tumor-localized DOX delivery with simultaneous photothermal ablation [29]. Reproduced with copyright permission from Elsevier, 2017. Legend: FA, folic acid; GO, graphic oxide; AuNPs, gold NPs.

Chauhan et al. [29] selected gold NPs (AuNPs) as the composite metal and folic acid (FA) as the graphene oxide surface functionalization moiety for active tumor targeting of the model anticancer drug DOX (see Figure 4). Yang et al. [30] have examined the uptake of graphene oxide which has been functionalized with PEG, noting that it has good solubility and biocompatibility. In addition to this, PEG-ylated graphene oxide, which accumulates in tumors and has low retention in the reticuloendothelial system, has also been reported [30].

Moreover, Huang et al. [31] have reported that the magnetic functionalized graphene oxide composite may be used as a nanocarrier for the delivery of anticancer drugs. Magnetic graphene oxide was produced by the authors by chemical co-precipitation of Fe_3_O_4_ magnetic NPs on graphene oxide nano-platelets. Furthermore, magnetic graphene oxide was modified by chitosan and mPEG-NHS through covalent bonds to synthesize mGOC-PEG. Irinotecan (CPT-11) or DOX was loaded to mGOC-PEG. Another experiment has been reported by Barahuie et al. [32] in which graphene oxide was synthesized in order to explore its potential use as a nanocarrier for an active anticancer agent, chlorogenic acid (CA). 

DOX-loaded PEG-ylated nanographene oxide (NGO) preparation has been reported by Zhang et al. [33] where this combination caused destruction of a tumor without recurrence. Qin et al. [34] have used FA-conjugated NGO-PVP as a carrier for DOX, where folate receptors of cancer cells possessed great affinity to bind with folic acid. The FA-NGO-PVP was delivered within the cytoplasm of folate receptor positive human cervical cancer cells (HeLa). The minimal cellular uptake for the nanocarrier was an indication of the adhering of the FA-NGO-PVP composites to the folate receptors on the surface of the A549 cells. Thus, the nanocarrier was shown to have photo-thermal sensitivity and could be used as an anticancer carrier for chemo-photothermal therapy. Furthermore, camptothecin (CPT) and DOX were involved in the sulfonated NGO sheets, where folic acid was attached to carboxyl groups of the sulfonate NGO sheets to deliver DOX. The cytotoxicity of FA-NGO/CPT/DOX to MCF7 human breast cancer cells has been highlighted elsewhere [35]. Zhao et al. [36] have also prepared pH-responsive chitosan/ graphene oxide nanohybrids to be used for the delivery of DOX anticancer. 

Liu et al. [37] have functionalized NGOs with PEG, using these to deliver the SN38 anticancer drug for colon cancer. Masoudipoura et al. [38] have reported a nanographene oxide conjugated with dopamine (DA-nGO) and laden with the anticancer drug methotrexate (MTX) to be supplied to positive human breast adenocarcinoma though adhering to DA receptors. GOs have demonstrated better characteristics when compared to CNTs, owing to their greater drug encapsulation ability. However, their use in biomedical applications is still limited because of their complicated synthesis and lower production yield, which makes them uneconomical.

#### 2.2.3. Nano-Diamond

Nano-diamond is considered to be another type of carbon-based nanomaterial and is rarely used in cancer treatment. ND is considered an allotrope of carbon with an average particle size between 4 to 6 nm and which in aggregation could be recorded as having particle sizes of 100 to 200 nm. The surface of each ND possesses functional groups which allow for a different series of active molecules to be conjugated to it, including anticancer drugs [39,40]. The appearance of functional groups on ND surfaces works in the form of a covalent bond between the ND and other material, such as a polymer, which improves the solubility of the ND powder in various solvents [39]. NDs have an excellent biocompatibility, meaning they can be used in chemotherapy. For example, the biocompatibility of a fluorescent ND powder with a particle size of 100 nm has been confirmed for kidney cells by Yu et al. [41].

Some trials which have reported the utilization of ND in biomedical applications are summarized below. Carboxylated NDs have been utilized to improve the solubility in aqueous media of some anticancer drugs. Purvalanol A and 4-hydroxytamoxifan, known to be effective drugs for breast and liver cancers, have been conjugated with NDs [42]. Moreover, efficient drug loading capacity has been confirmed for NDs with negative charge which are accompanied by both NaCl and cationic DOX ions to achieve better treatment for HT-29 colorectal cancer via a more efficient method, in comparison to other carbon-based materials [43].

Upal et al. [44] have studied, using *invitro* testing, modified and surface-unmodified (–NH_2_ and –COOH) ND for its ability to carry the anti-HIV-1 drug efavirenz and its cytotoxicity. It was found that there was a highly significant drug loading capacity for the unmodified ND conjugated drug formulation in comparison to the surface-modified ND, which had a very low toxic effect. NDs have shown few successful trials on an in vitro basis but animal studies need to be carried out in the near future to figure out their effect on the vital organs and their clearance mechanism. However, NDs suffer from the same restrictions as carbon-based materials, as mentioned above.

### 2.3. Mesoporous Silica Nanoparticles (MSNPs)

**Figure 5:**
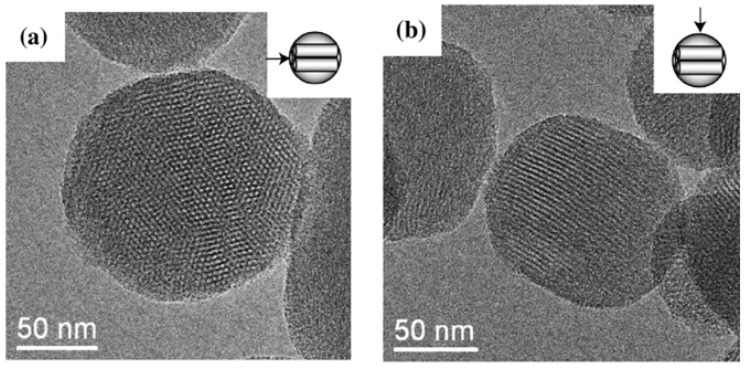
TEM images of mesoporous silica nanoparticle (MSNP) material recorded from the direction (**a**) parallel or (**b**) perpendicular to the long axis of the meso-channels [45]. Reproduced with copyright permission from Elsevier, 2008.

One common types of NP which is used as a drug delivery carrier is the solid mesoporous silica nanoparticle. Mesoporous NPs are particles in which the core of the particle is solid in nature while the shell or the particle’s surface is porous in nature. Alternatively, the particle itself is porous in nature. MSNPs are inorganic particles comprising of a honeycomb-like porous structure with empty channels (pores) of nanosized dimensions [45]. There are two types of mesoporous silica according to their particle size: mesoporous silica microspheres and mesoporous silica nanospheres. Although the use of mesoporous silica microspheres is important in numerous non-biological applications, they are not appropriate for use in many biomedical applications. However, the average particle diameter of a typical MSNP is ~100 nm with pore volumes ~0.9 cm^3^/g, pore volume surface areas ~900 m^2^/g, and pore sizes ~2 nm [46]. A hexagonal crystal structure has been recorded for MSNP porous channels (Figure 5).

Slowing et al. [47] have studied the implications of surface functionalization of MCM-41-Type MSNPs on the mechanism and efficiency of endocytosis different charge profiles on HeLa. The study concluded that the ability of MSNPs to escape endosomal entrapment was influenced by surface functionalities as this is considered to be a key factor in the designing of compounds that are effective for intracellular delivery.

### 2.4. Gold Nanoparticles (GNPs)

Gold nanoparticles are considered excellent carriers for anticancer drug delivery because of their many unique features, such as their tunability in surface characteristics and particle size, their inertness, and their biocompatibility. Sabu et al. [48] have demonstrated that gold NPs can be loaded with anticancer agents. Paclitaxel (an anticancer drug) has been conjugated covalently by Gibson et al. [49] to gold NPs of size 2 nm, and the system has been found suitable to be used as a self-therapeutic. Dhar et al. [50] have conjugated gold NPs with DNA (producing DNA-AuNPs) to be employed as carriers of platinum compounds for the treatment of cancer. Cellular internalization of the tethered platinum was more effective than free cisplatin in killing cancer cells.

Brown et al. [51] have reported that oxaliplatin in its active form conjugated with gold NPs has possessed an effective killing ability with regard to cancer cells compared to pure gold NPs. It has also been proven that BSA-capped gold NPs can effectively be used as a nanocarrier for anticancer drug MTX and has been considered to be an anticancer for MCF-7 breast cancer cells when compared to non-capped NPs, as earlier declared by Yu-Hung et al. [52]. Furthermore, core/shell gold NPs have been fabricated and evaluated in order to be used as carriers for dual treatment where a radiosensitizer was presented by GNPs and the anticancer drug (DOX) [53].

Manivasagan et al. [54] have demonstrated a new method for the production of gold NPs to be used as a drug carrier or to be useful in photo-acoustic imaging (PAI). In particular, they have synthesized multifunctional doxorubicin-loaded fucoidan-capped gold NPs (DOX-Fu AuNPs). Dhamecha et al. [55] have also reported that GNPs work as an effective drug carrier for targeting DOX to fibrosarcoma cancer cells. They have been observed to subsequently reduce DOX-associated cardiac toxicity and myelo-suppression. DOX was seen to be absorbed onto GNPs with a high drug loading capacity, and industrial scalability of the process was demonstrated in their research [55]. Scientists working with GNPs face very challenging issues related to their removal from the human body along with their accumulation in the liver and kidney.

### 2.5. Other NPs Used as Drug Delivery Carriers

Some other inorganic materials (Table 1) have been used as drug delivery systems, and include functional multilayer fluorescent nonporous silica SNPs with an external shell, e.g., those containing primary amino groups [56]. AL-Ajmi et al. [57] have also prepared crystalline ZnO NPs using organic precursor techniques. These ZnO NPs were used as a drug carrier to deliver 5-Fluorouracil (5 Fu).

## 3. Inorganic NPs for Hard Tissue Regeneration

The ideal nanostructured composite presented in the human is bone, which is considered the typical model of a hierarchical microstructure [58]. Nonstoichiometric nanocrystalline hydroxyapatite (HAP), (Ca_10_(PO_4_)_6_(OH)_2_), which is 20–40 nm in length, is the major inorganic component of bone, and Type-I collagen, which is ~300 nm in length, is its major organic counterpart. It has been revealed that HAP nanocrystals cover bone collagen molecules to form nanocomposites fibers. HAP C-axes are generally aligned along the collagen molecules in these nanocomposites [59]. 

**Figure 6:**
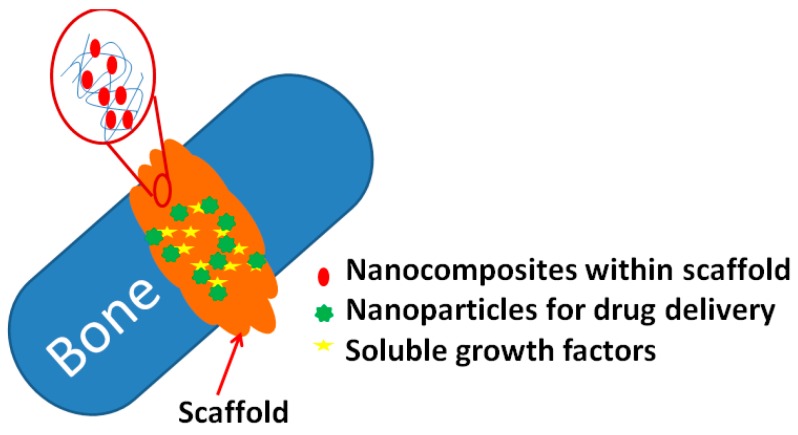
NPs used to repair a bone fracture: in cases of bone fracture, nanomaterials have been implanted into the target area (adapted from [62]).

Recently, the synthesis of nanomaterials for bone repair has been explored based on the simulation of various bone properties. One significant characteristic of materials used for bone regeneration is their mechanical property, and this property is possessed by different nanomaterials [60,61]. The other approach is to carry out surface modification at the nano-level, as this can provide an improved matrix for osteoblasts to grow and function (Figure 6) [62]. Several types of materials are suitable as biocompatible materials for bone healing with applications possible for metallic oxides (aluminium oxide, zirconium dioxide, and titanium dioxide), the CaPs family (hydroxyapatite, tricalcium phosphate (TCP), and calcium tetraphosphate) and glass ceramics (bioglass and ceravital) [63].

In addition, nano-scaffolds have superior properties in comparison to micro-scaffolds, owing to their higher porous microstructures that can mimic a real extracellular matrix (ECM), their porosity, and their other physical properties. This in turn improves their osteoblast adherence and viability [60,64,65]. Moreover, NPs enable the delivery of drugs and growth factors to promote healing and functional recovery. Modifying the surface of these biocompatible materials at the nano-scale for bone healing purposes has also been studied [66].

### 3.1. Carbon Nanotubes

Owing to the impressive mechanical characteristics of carbon nanotubes, the idea of reinforcing brittle ceramic such as HAP using CNTs for the treatment of bone disease and fractures has been considered in the last three decades. Zhang and Kwok [67] have investigated the bone healing ability of HAP-TiO_2_-CNT NPs. They noted the formation of an apatite layer on the surfaces of monolithic HAP and HAP-CNT and HAP-TiO_2_-CNT NP coatings after 4 weeks of soaking in Hanks’ solution. In addition, no remarkable effect was recorded on the formation of the apatite layer on the surfaces of TiO_2_ and CNT when added to HAP. Furthermore, it has been reported that the addition of CNTs to HAP has improved the mechanical properties of the final composite as recorded by nano-indentation technique. These results revealed that the higher the addition of CNTs the greater the mechanical properties which were achieved compared to pure HAP [68].

A different application for the CNTs that has been reported aside from their being used as reinforcing material during bone regeneration is as a biosensor for bone regeneration [69]. Using NPs as a delivery system for bio-active molecules from bone healing, for example, both genes and proteins can be delivered to enhance propagation of osteoblasts, angiogenesis, and a reservoir for the necessary calcium salts [69]. Furthermore, one study has been conducted using multi-walled carbon nanotubes (MWCNTs) impregnated into HAP and the dip coating of a nanocomposite onto a titanium alloy (Ti-6Al-4V) plate to enhance the surface roughness of the implant. The authors noted that the mechanical properties of the HAP coating were enhanced and the surface roughness was reduced upon the addition of low concentrations of MWCNTs in comparison with pure HAP. Moreover, normal cell attachment and the growth process were confirmed by cell studies [70].

Other studies have discussed the cytotoxicity of titanium alloy implants coated with plasma-sprayed CNT-reinforced HAP embedded in rodents’ bones, and it has been observed that there is no adverse effect or cytotoxicity associated with the addition of CNTs to bone tissues [71]. Balani and Agarwal have also confirmed the non-toxicity of a HAP-CNT coating on Ti-6Al-4V implants and have observed the ultimate growth of human osteoblast cells near the CNT regions [72].

**Figure 7:**
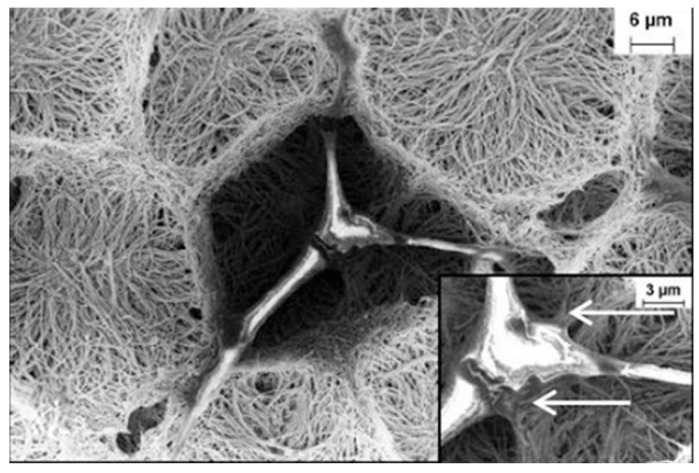
Scanning electron micrographs of L929 mouse fibroblasts growing on a multi-walled carbon nanotube (MWCNT)-based network [73]. Reproduced with copyright permission from the American Chemical Society, 2004.

CNT-based 3D networks have been found to be suitable for cell growth as a biomatrix scaffold [73]. In [73] a 3D network was produced by exerting chemically-induced capillary forces in order to manipulate the vertically aligned CNT array and the authors found that those networks seemed to be suitable for cell growth as a scaffold (Figure 7).

Another scaffold based on MWCNTs has been reported by Abarrategi et al. [74]. Here, the MWCNTs were functionalized by chitosan (MWNT/CHI) and these nanocomposites were affirmed to be a scaffold for tissue engineering. In addition, this study showed that the MWNT/CHI scaffolds implanted subcutaneously accompanied by recombinant human bone morphogenetic protein-2 (rhBMP-2) for 3 weeks had increased the growth of C_2_C1_2_ cell lines. Afterwards, the MWNT/CHI scaffold composite was replaced by cells and bone regeneration was observed. In this study the authors confirmed the suitability of the MWNT/CHI scaffolds for utilization in bone reconstruction. Apart from hard (bone) tissue regeneration, CNTs have also been utilized for soft tissue regeneration (nerve) in order to assess cells behavior [75]. For this purpose, Keefer et al. [76] designed a neural network made of tungsten and stainless-steel wires and the electrode’s wires were coated with CNTs by electrochemical methods.

### 3.2. Titanium Dioxide (TiO_2_)

Titanium dioxide or titania (TiO_2_) has been used as a bioactive coating and there has been consideration given to using TiO_2_ within the coating as reinforcement as a technique for improving the mechanical reliability of HAP. Also, it has been proven that TiO_2_ has the ability to induce osteoblast cell adhesion and growth [77,78]. It has been determined that the smaller the titanium grain size the greater the osteoblast adhesion. This may be because of the higher surface area attributed to the TiO_2_ nanoparticles. Various techniques have been used to improve the TiO_2_-HAP nanocomposite coatings for biomedical applications in order to enhance the strengths of coating and adhesion, reducing a loss of implants because of potential failure at the metal-coating interfaces and achieving an increase in biological response [79,80].

Kuwabara et al. [80] tested the bone formation ability on TiO_2_-HAP nanocomposite coatings with a thickness of 100 nm. They cultured the osteoblast cells, which were derived from rat bone marrow, on the surface of coatings with different electrical charges and found that the HAP- and TiO_2_-coated surfaces demonstrated a higher influence with regard to adhering to and propagation of cellsin comparison to pure HAP or pure TiO_2_-coated surfaces. Ahmed et al. [81] revealed that the proliferation of mesenchymal stem cells (MSCs) was enhanced after using a Se/Ti nanocomposite. Furthermore, another study has unveiled a new composite which was generated using HAP/chitosan bioactive nanocomposites [82].

## 4. Bio-Imaging

Due to the magnificent properties of metal inorganic NPs, they have been directed to plenty of biomedical applications, especially in the bio-imaging field. The size of NPs plays an important role in the bio-imaging process, as NPs are preferred to other small molecules owing to their fast penetration in biological tissues and their ability to pass through the circulatory system. In addition, NPs exhibit limited renal excretion and prolonged blood circulation time, which allows repeated passing of NPs through tumors’ vessels. Unlike organic NPs, inorganic NPs exhibit inefficient extra vasation inside tumors, which involves inorganic NPs tending to remain in the vasculatures of tumors without being in the interstitial spaces. This reduces the marking of nonspecific tumor cells within the imaging spaces [83,84,85,86].

An important approach for improving the imaging process which has been conducted by researchers has been to load one NP with different contrast agents related to several imaging techniques for multimodal imaging which attempts to overcome the limitations related to single imaging techniques [87,88]. Moreover, imaging and treatment can be achieved by the same NP [89,90]. NPs such as semiconductor quantum dots (QDs) have been considered for photoluminescence and have been widely used in bio-imaging [88,89,90]. In addition, luminescent UCNPs and SERS nanoprobes based on gold and silver NPs can be used for bio-imaging [91,92,93]. Magnetic NPs with sizes 1–100 nm can display superpara-magnetism meaning they can be widely used as a contrast agent in magnetic resonance imaging (MRI); iron oxide magnetic NPs coated with dextran can also be used in MRI [94,95]. Because of the optical properties of single-walled CNTs, such as high optical absorption and photoluminescence in the near IR region and strong resonance Raman-scattering, single-walled CNTs are widely utilized for bio-imaging [96].

### 4.1. Quantum Dots

**Figure 8:**
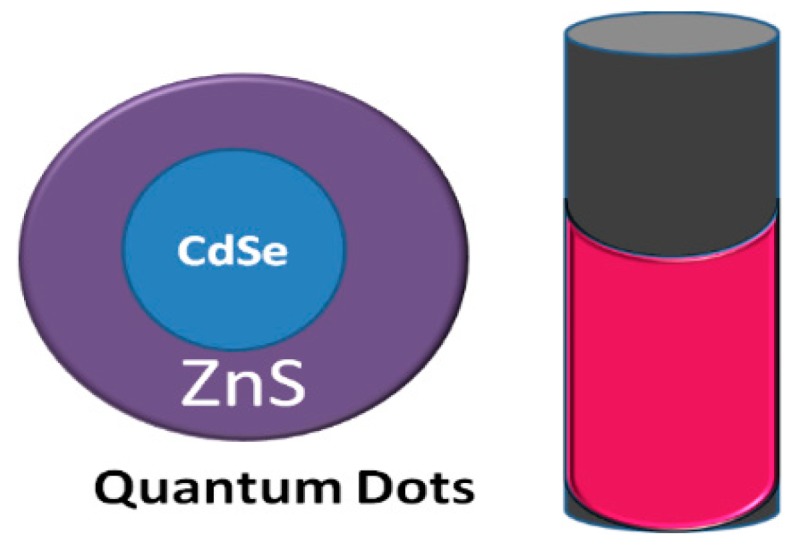
A semiconductor quantum and a quantum dot (QD) aqueous solution under UV light showing bright pink fluorescence. QDs are widely used in fluorescence imaging (adapted from [99]).

QDs are NPs composed of semiconductor materials or atoms from groups II-IV or III-V, includingcds, cdse, cdte, zns, znse, zno, GaAs, InAs, and InP, owing to their unique optical properties. Each semiconductor material is covered by another semiconductor material, and these have a large spectral bond-gap which allows for an increase in the photo stability and quantum yield for the emission process; QD NPs show stability against an aggregation with capping agents [97]. In addition, these materials have a high molar extinction coefficient and high absorption from UV to near IR [88], where the changing diameter of NPs can modulate the excitation and emission peaks of QDs, with QDs showing sharp emission peaks that are considered ideal for multi-color imaging [97,98,99] (Figure 8). 

Cai et al. [98] have reported the in vivo integrin αvβ3 imaging of RGD peptide-conjugated QDs. Their results revealed that RGD peptides when conjugated with PEG-ylated QDs demonstrate maximum emission at 705 nm when injected intravenously into mice bearing U87MG tumors. In addition, the results showed a tumor contrast 20 min after injection and reached a maximum 6 h from injection. Another in vivo study conducted by Chen et al. [87] attempted to use optical and polyethylene terephthalate (PET) imaging of VEGFR in vasculature tumors by using QDs, where, they reported that, the amine-functionalized QDs conjugated with VEGF protein and then were exposed to radiation to be radio-labelled for VEGFR-targeted NIR fluorescence and PET imaging of tumor vasculatures. Furthermore, Ostendorpet al. [93] have utilized cyclic Asn-Gly-Arg (cNGR), which was seen to conjugate with paramagnetic QDs (pQDs) as a tumor nanoprobe, where cNGR targeted (the aminopeptidase N) CD13 on the endothelium of a tumor and was used for fluorescence/MRI dual evaluation of tumor activity.

Yong et al. [89] prepared InP-ZnS QDs that were used for in vivo detection of pancreatic cancer because of the low cytotoxicity exhibited by InP-ZnS. These were considered to be biocompatible nanoprobes for diagnosis of pancreatic cancer cells. The results showed that in primary and metastatic pancreatic cells the antigen receptors for anti-claudian 4 were over-expressed and QDs conjugated to anti-claudian 4; in addition, a polyclonal antibody was conjugated to QDs and no damage to the cells was observed. However, Cai et al. [98] have explored PET imaging of radio-labelled QD-RGD, which showed predominant uptake of QDs in the spleen and liver. Another problem related to QDs could be their presence within the body, where long retention time for accumulation of QDs has been observed. There is in vivo toxicity related to the presence of II-IV groups inside the body, but there are strategies to overcome this problem where there is a new generation of QDs that has been developed to reduce toxicity, including Cd-free QDs. Repeated demands of the product development of QDs and their related fields should be considered for their side effects and in the long run, substantiated by prototype modules and pilot-line fabricating, especially in light of arrangement producers and subsidizing organizations frequently setting their needs in relation to being dependent on these demands.

### 4.2. Carbon Nanotubes

**Figure 9:**
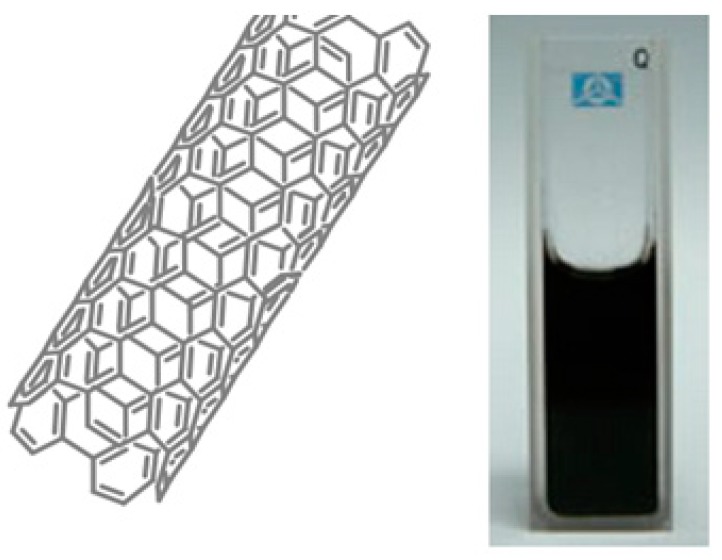
A single-walled carbon nanotube and an aqueous solution of SWNTs functionalized by PEG-SWNTs with highly optical properties, which are considered excellent platforms for biomedical imaging [99]. Reproduced with copyright permission from Springer Nature, 2010.

As mentioned above, there are two types of CNTs, namely, SWNTs and MWNTs. SWNTs have strong optical absorption in the visible and NIR regions due to the optical properties of CNTs, such as resonance. Raman-scattering and photoluminescence in the near IR region make CNTs useful for biomedical imaging. SWNTs exhibit different optical absorption peaks from the UV region to the near IR region, which made these NPs suitable for utilization as photo-thermal agents and photo-acoustic imaging agents [99] (Figure 9).

Liu et al. [100] have reported how in vivo integrin αvβ3 imaging was able to utilize SWNTs and SWNTs functionalized by PEG-ylated phospholipids to increase water solubility which was labelled by a 64-Cu radioisotope for micro PET imaging. RGD peptide conjugated 64 Cu radiated SWNTs to PEG coating (SWNT-PEG 5400-RGD) was injected intravenously into the glioblastoma U87MG tumor in mice and then monitored by a micro PET. The results demonstrated higher uptake of SWNT-PRG5400-RGD by the tumor (~13% of dose ID/g) when compared to the tumor uptake of SWNTs without combination with RGD (4~5%) of injected dose per gram tissue. Moreover, Smith et al. [97] have used RGD conjugated with PEG-ylated SWNTs as a Raman nanoprobe for in vivo tumor imaging in mice, where SWNT-RGD was injected intravenously into the mice’s tumors with high integrin αvβ3 expression. Raman microscopy showed strong signals for tumor cells injected with SWNT RGD while weak signals were recorded for the tumors injected with non-targeted SWNTs. They also utilized SWNT-RGD as a contrast agent for tumor photoacoustic imaging. PET imaging and Raman imaging showed strong photoacoustic signals in U87MG tumors. CNTs with their impressive biocompatibility have demonstrated great success in the imaging field, thus encouraging scientists to explore their applicability in the diagnosis of most common diseases other than cancer. Nevertheless, CNTs have not been explored widely owing due to their difficult and expensive preparation methods.

### 4.3. Super Paramagnetic Iron Oxides NPs

Superparamagnetic iron oxide (SPIO) NPs have been widely utilized as a contrast agent in MRI imaging; FeCo alloys have the best magnetic properties but because of oxidation and toxicity their appearance in biomedical applications has been limited [101]. Iron-based oxide NPs have been investigated by Won et al. [95], in which coated FeCo nanocrystals with a single layer of graphite carbon (GC) was used as a contrast agent for MRI. These NPs were PEG-ylated to increase the solubility of the FeCo/GC NPs. The water-soluble FeCo/GC NPs exhibited higher relaxivities on a per metal atom basis compared to other materials which utilized MRI as a contrast agent. Afsaneh et al. [102] have reported on recently developed MRI and PET imaging probes using SPIO NPs as a contrast agent where iron oxide NPs were coated with various coats, such as poly aspartic acid, PEG, dopamine, and dextran. The effects of these coats were discussed with regards to the particle size, targeted organ, final uptake, and time retention in the studied organ.

One of the methods by which magnetic NPs are delivered to the diseased organ is by intravenous infusion or by means of a blood circulatory system. Another method relies on utilizing magnetic nanoparticles suspensions for infusion. A steady uniform solution is required to avoid the aggregation of the NPs. Particle size and surface functionalization are two parameters required for the stability of the magnetic colloidal suspension. Determination of appropriate magnetic nanoparticles is the principal significant advancement for bioapplication. For applications in drug delivery, the magnetic nanoparticles are required to be steady in water at neutral pH, which relies on their size, charge, and surface functionalization. However, super magnetic materials, for example, cobalt and nickel, are not utilized in biomedical applications because of their lethal properties and oxidation susceptibility.

### 4.4. Gold NPs

Gold NPs have been highlighted among contrast agent materials for their bioimaging application. Monodispersity, stability, and higher attenuation coefficients for X-rays are the impressive properties which make these NPs suitable for this process. Peng et al. [91] have found that a way to synthesize dendrimer-stabilized gold NPs is by use of amine-terminated fifth-generation poly(amidoamine) (PAMAM) dendrimers. These were modified by diatrizoic acid as stabilizers for enhanced CT imaging. Li et al. [103] prepared Au-coated iron oxide (Fe_3_O_4_-Au) nanoroses to be used as a probe for multi-function as well as aptamer-based targeting, MRI, optical imaging, photo thermal therapy, and chemotherapy. Zhang et al. [104] prepared PEG with PEI-stabilized gold NPs for blood pool, lymph node, and tumor CT imaging. Moreover, Yigit et al. [105] synthesized gold NPs conjugated to 3,3-diethylthiatricarbocyanine iodide (AuNP-DTTC) which is used as a contrast agent for in vivo MRI and Raman spectroscopy. Here the probe consisted of MRI-active super paramagnetic iron oxide NPs coupled with AuNP-DTTC. Tailoring the properties of gold NPs and utilizing chemical techniques has grown extensively in the last two decades, especially in the field of bio-imaging. Developed tests and probing techniques rely on advances in gold nanoparticles conjugates, new optically-controlled functional materials, new highly specific color-coded probes of cellular function, and new optically-based therapeutic methods.

## 5. Porous Membranes

**Figure 10:**
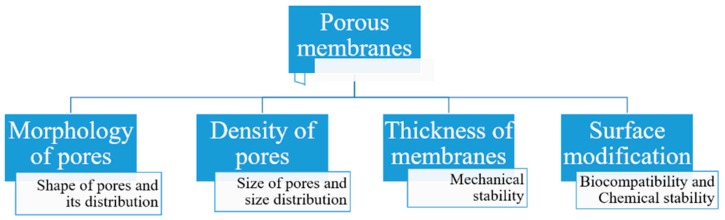
Key characteristics of porous membranes.

The second objective of this review is to present an overview of the therapeutic applications of porous membranes and their key challenges. Porous membranes have been used in numerous engineering applications such as molecular separation, catalysis, and filtration, etc. [106,107]. Literature shows that porous membranes are frequently regarded as nanoporous structures because the pore size of such membranes lies between 1 and 100 nm, although several terminologies have been used to explain these porous membranes and the terms nanoporous and microporous are used based on pore size [108,109]. In this section, different types of membranes, types of materials, etched membranes, and the fabrication of micro- and nanoporous membranes are briefly explained (Figure 10). In the following sections, the properties, surface modification techniques, biocompatibility, and drug delivery applications are also discussed. Lastly, we describe the key challenges and future prospects of these membranes.

### 5.1. Membrane Classifications

Membranes can be classified based on the following characteristics.

#### 5.1.1. Material

Membranes comprise of inorganic, organic, polymeric, and composite materials. Mostly, the organic/polymeric membranes are made of materials such as polycarbonate (PCL), polyethylene terephthalate (PET), and polysulfones, etc. (Table 2). These composite materials contain two different materials and combine apolymer and an inorganic material (e.g., a polymer with a ceramic). They are developed in particular to improve the stability, permeability, and selectivity [110]. 

#### 5.1.2. Size, Shape, and Order of Pores

Membranes are classified according to their size, distribution of size, order, and shape. Hence, membranes pores<2 nm are called microporous, those with pores 2–50 nm mesoporous, and those with pores>50 nm macroporous. The shape of the pores can be cylindrical, slit-like, conical, or irregular in shape. The pores should be well arranged in a vertical order instead of in a tortuous network [111]. The porous network for drug delivery depends upon the following properties:

- Optimum pore size with narrow distribution;
- Less flow resistance to allow high flux;
- Sufficient mechanical strength with adequate chemical and thermal stability, and;
- Biocompatibility.

#### 5.1.3. Fabrication Method

There are various methods available for fabricating membranes and they include ion-track etching, lithography, selective electrochemical leaching, focused ion beam etching, the sol-gel process, and the phase separation technique [111,112].

##### Ion-Track Etching

The polymeric membrane plays an essential role in drug delivery. One of the most common methods applied for formulating polymeric membranes with ordered pores is ion-track technology. This process is achieved by exposing a thin film of polymer with heavy ions which form ion tracks. The ion tracks are allowed to form pores via a chemical etching process which selectively attacks the ion tracks. The pores formed by this process are cylindrical or conical in shape with a diameter from 10 nm to within the µm range. The limitation of this process is that manufacturing of pores of a diameter less than within the nanometer range is not possible [113].

##### Micromolding

Another important method used to prepare membranes is micromolding. This technique is similar tothephase separation method and involves a solution of the polymer being poured into a mold, which is solidified by the phase separation process. Langley et al. and Ultricht et al. have discusseda porous structure developed by a phase separation micromolding technique which considers the structural, physical, and chemical properties of the polymers used [109,110]. 

##### Lithography

Micro/nanofabrication technologies have been recently developed as the most interesting methodology used to produce an ordered array of micro/nanopores on the surfaces of silicon. Conventional methods have drawbacks such as broad size distribution of pores, poor mechanical strength, and poor chemical stability. Micro/nanofabrication techniques are widely used to overcome these problems. One example is the work of Desai et al. in which biocapsules with controlled pores of about 7 nm were developed. The authors established that these nanoporous membranes allow exchange of nutrients, waste products, and therapeutic proteins, and that they are biocompatible, providing immune support to cells. In addition, they used their fabricated membrane for an implantable pancreas and oral drug delivery formulation [114,115]. 

##### Focused Ion Beam Etching

Polymeric membranes with a well-ordered array of pore structures and a size range of about 100 nm with a thickness of 1–5 µm have been able to be prepared. The membranes formed with this technique are called nanosieves and are also effectively achieved by photolithography. An excellent example of this method has been prepared by Tong et al. which has included an ordered structure of a cylindrical membrane of diameter 25 nm [116].

##### Electrochemical Etching

The most extensively studied membrane-producing electrochemical etching method is the anodic membrane technique. The membranes fabricated with this technique produce a honeycomb-like and ordered structure. The pore morphology and size can be controlled through the process of anodization [117]. The organization of the porous structure depends on the voltage and chemicals used in the fabrication. These ordered channel assemblies of membranes are obtained by an anodization process. The most effective technique for preparing well-arranged porous membranes with large dimensions involves this process. During the anodization procedure, thin ordered structures are engraved within a larger dimension membrane [118]. Within the small ordered structures biomolecules are encapsulated, and these membranes are used for diffusion-controlled delivery systems. The drug molecules are encapsulated inside the micelles as biocapsules and it has been shown that membranes containing biocapsulesare able to release the drugs in a controlled fashion [119]. 

#### 5.1.4. Surface Modifications

When membranes come into contact with physiological fluids, three main processes occur which create problems, namely, biofouling, degradation of the membrane, and immune reactions caused by the membrane. There are various approaches to addressing these problems, and these are discussed as follows. If cells, proteins, and other materials accumulate on a membrane surface when the membrane is in contact with a biological environment, a process is occurring which is called biofouling [106,120]. The second issue is that this also causes tissue encapsulation, which leads to fibroblast proliferation, collagen synthesis, and proliferation of blood vessels. These processes in turn lead to vascular tissue capsule formation, which delay the transport of glucose molecules in biological environments due to steric hindrance [121]. Wound healing occurs via the processes of hemostasis, inflammation, and formation of scars and repair. During the implantation of the membrane, cells of epithelial connective tissue and the basement membrane may be damaged. Firstly, the bruised area is filled with coagulated blood, and this clotting allows neutrophils. The day after wound injury, the inflammatory cells disturb the function of the membrane by taking nutrients and releasing proteolytic enzymes and free radicals. On the third day, there is occurrence of macrophages and granulation tissue in the wounded area, followed by neovascularization on fifth day. Finally, at the end of the first month, the formation of a mature scar in the epithelial layer of the tissues is observed. Hence, the biocompatibility depends on the surface of the implanted membrane, which influences each stage of the process. A thick vascular fibrous scar formation leads to diffusion of analytes and uptake of nutrients, resulting in reduced response of the membrane [115,122].

In order to reduce biofouling, several reports have demonstrated the use of coatings and other methods of surface treatment. For this, the membrane surface is treated with cross-linked polymers of poly (hydroxyethyl methacrylate) or poly (ethylene glycol). Polymer coatings on the membrane surface result in electrically neutral, polar, flexible, and swellable membranes in water, and create an interface between the membrane surface and the physiological environment. Coatings of poly (hydroxyethyl methacrylate) or poly (ethylene glycol) are considered the most attractive for membranes because water-soluble drugs diffuse through the water-swollen polymer layer [123,124,125].Biocompatibility of the implantable membrane is characterized by inflammatory responses. Surfactants are also called surface-active agents and have hydrocarbon tails attached to polar head groups. Many membranes used for drug delivery contain these surfactant molecules as a plasticizing agent. Unexpectedly, these surfactants diffuse out of the membranes and to the surface, until they get depleted. Accordingly, these agents need calcium chelating plasticizers which limit the enzyme in the coagulation cascade [126,127]. Keeping in mind the characteristics of the membrane and the physiological environment, the biocompatibility of the material should also be addressed. In addition, it is very essential to prove that there is very little or no leaching, degradation, biofouling, and inflammatory responses. Therefore, selection of the perfect surface coating is important to figure out problems in a biological environment [128]. 

### 5.2. Drug Delivery by Membranes

The use of membranes to deliver drugs/bioactives is opening up new therapeutic advantages like increasing the solubility of bioactives, protecting bioactive molecules from degradation, providing a sustained release of the actives, improving drug bioavailability, providing targeted delivery, decreasing lethal effects, offering an appropriate form for all routes of administration, and allowing for rapid formulation development. Moreover, they can carry one or more bioactive agents and have been developed into different classes of carriers. The different carriers can be carbon-based nanomaterials, polymeric membranes, and inorganic membranes [129]. 

#### 5.2.1. Polymeric Membranes

**Figure 11:**
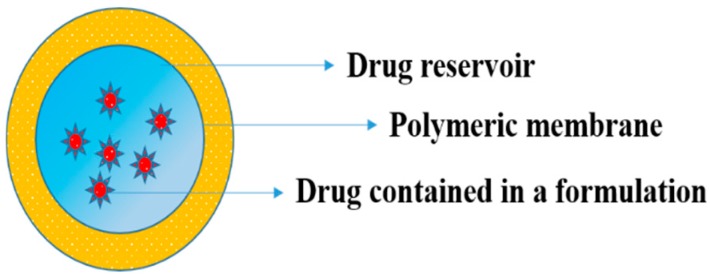
Polymeric membrane used for drug delivery system.

**Figure 12:**
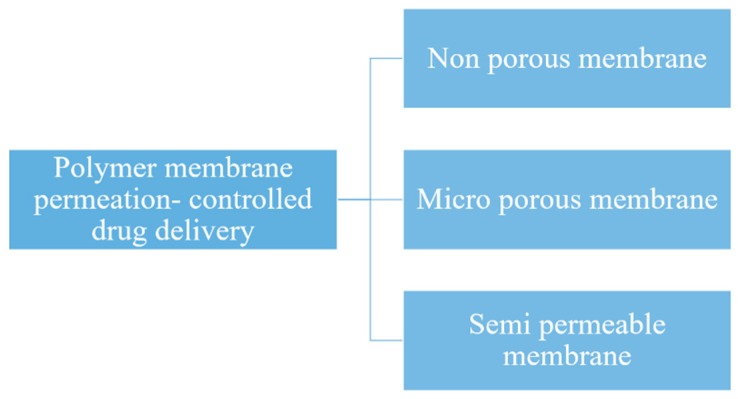
Classification of polymeric membranes for drug delivery.

Here, the drug formulation is encapsulated in the compartment of a drug reservoir, in which the surface of the drug-releasing layer is covered by a rate-controlled polymeric membrane. The drug reservoir could be solid, a solid dispersion, or a drug solution in liquid form (Figure 11). The encapsulation process for preparing the drug formulation inside the reservoir compartment includes fabrication by microencapsulation, coating, and molding techniques. The polymeric membrane is manufactured from a nonporous, microporousor semipermeable membrane (Figure 12) [130].

Drug release from the polymeric membrane must be at a constant rate (Q/t) which is well-defined by the Equation (1),

(1) $$\frac{Q}{t}=\frac{K_{m/r}K_{a/m}D_{d}D_{m}}{K_{m/r}D_{m}h_{d\;}+{\;K}_{a/m}D_{d}h_{m}}{\;X\;C}_{R},$$

where, K_m/r_ and K_a/m_ are the partition coefficient of the drug molecule from the reservoir to the rate-controlling membrane and from the membrane to the aqueous layer, respectively, D_d_ and D_m_ are the diffusion coefficient of the rate-controlling membrane and the aqueous diffusion layer, respectively, h_m_ and h_d_ are the thickness of the rate-controlling membrane and the aqueous diffusion layer, respectively, and CR is the drug concentration in the reservoir compartment. The drug release from this system is controlled at a preprogramed rate by controlling the partition coefficient, diffusivity of the drug molecule, the rate-controlling membrane, and the thickness of the membrane. There are several controlled release polymeric membrane drug delivery systems which have been successfully marketed and some of the examples of these are outlined below [131].

#### 5.2.2. Transdermal Systems

**Figure 13:**
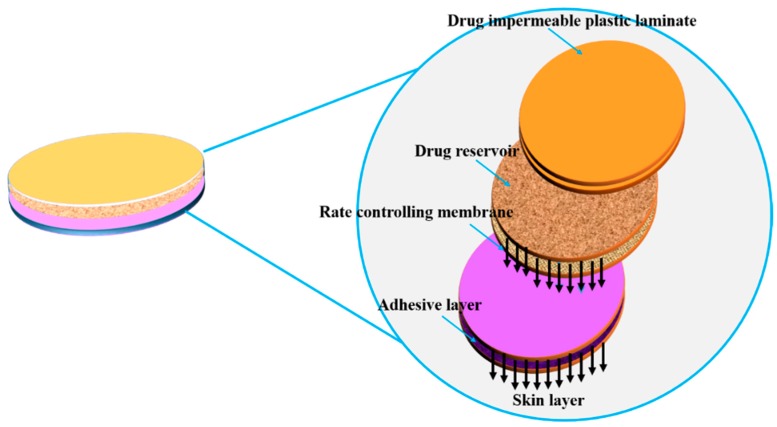
Diagrammatic representation of membrane permeation-controlled system in which the drug reservoir is sandwiched between the membrane layers and the adhesive layers facing the skin’s surface (adapted from [134]).

In this type of system, nitroglycerin and lactose are triturated in silicone fluid which is then encapsulated in a thin membrane of ellipsoid shape. The reservoir of the drug layer is introduced in between the metallic plastic laminate (backing sheet) which is impermeable and ethylene-vinyl acetate copolymer porous membrane which is the rate controlling membrane. This is formulated by the injection molding process and a thin layer of adhesive (silicone) is again coated on the permeable drug membrane for the immediate contact of the drug-releasing membrane with the skin’s surface to be maintained and achieved (Figure 13). Nitroglycerin (0.5 mg/cm^2^)/day is delivered transdermally for treating angina [132]. Other examples are Estraderm-controlled delivery of estradiol for 3–4 days to relieve postmenopausal syndrome and osteoporosis [133], Duragesic-controlled delivery of fentanyl for 72 h to relieve chronic pain [134] and Androderm-controlled delivery of testosterone(24 h) used as an additional therapy for patients with testosterone deficiency [135].

#### 5.2.3. Ocusert

**Figure 14:**
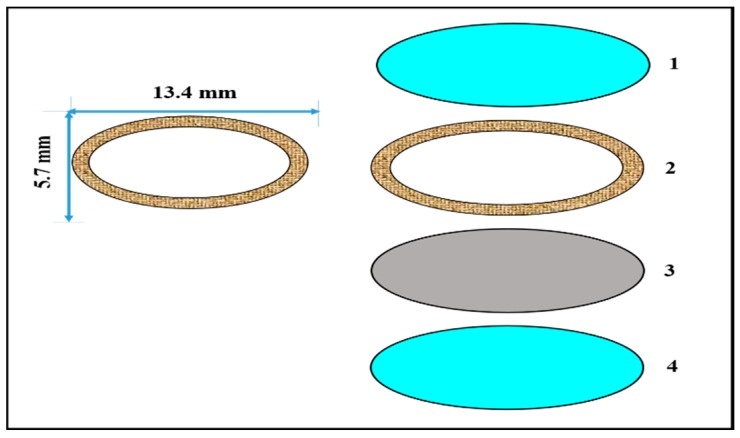
Ocusert: (1) and (4) show transparent polymer membranes, (2) shows a titanium dioxide white ring, and (3) shows a pilocarpine core reservoir (adapted from [136]).

In this type of system, in between two transparent microporous membranes a drug reservoir layer containing a pilocarpine-alginate complex is fabricated (Figure 14). The porous membrane permits the tears to permeate into the layer containing the drug. When the microporous membrane comes into contact with the tear fluid, it will dissolve and constantly deliver the pilocarpine at a rate of 20 to 40 mcg/h for treating glaucoma disease [136].

#### 5.2.4. Progestasert

**Figure 15:**
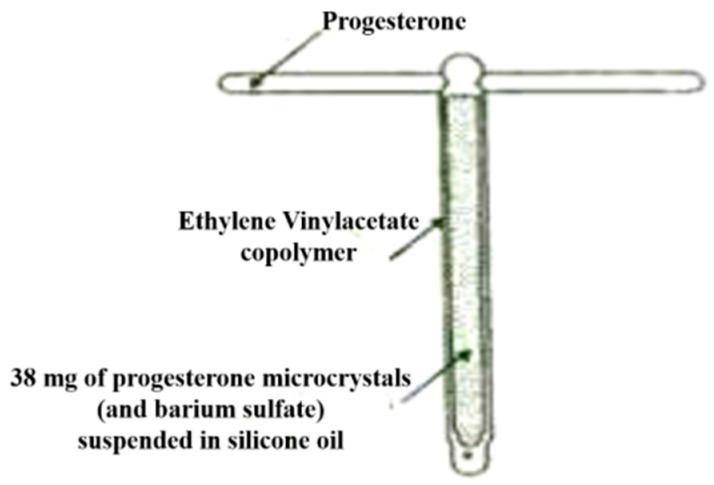
Progestasert IUD with structural components shown (adapted from [137]).

In this type of system, a drug reservoir layer is used which consists of BaSO_4_ and progesterone dissolved in silicone fluid and encapsulated into a T-shaped device using an ethylene-vinyl acetate copolymer (nonporous membrane) (Figure 15). This system is particularly designed to continuously deliver progesterone to the uterus with a dose of 65 μg/day to attain contraception for a year [137]. Another example of this system is the Mirenaplastic T-shaped device(steroid reservoir), which contains 52 mg of levonorgesterol for achieving contraception for 5 years [138].

#### 5.2.5. Polymer Matrix Diffusion-Controlled Drug Delivery

**Figure 16:**
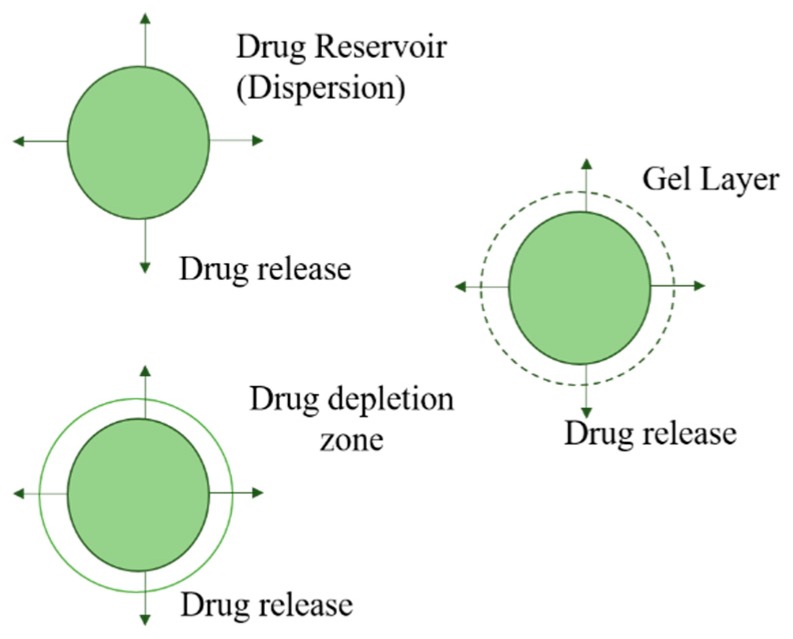
A polymer matrix diffusion-controlled system.

In this type of pre-programmed delivery system, the drug reservoir is prepared by homogenous dispersion of the drug in a rate-controlling polymer matrix fabricated by a hydrophilic or hydrophobic polymer. Secondly, the drug dispersion in the matrix is fabricated by blending the drug particles with a polymer or highly viscous base polymer followed by the cross-linking of polymer chains. Next, the drug solids are mixed in a rubbery polymer at an elevated temperature. The resulting drug-polymer dispersion is then extruded or molded to form a drug delivery device of varying shapes/sizes (Figure 16). It can also be prepared by dissolving the drug and the polymer in a solvent, followed by solvent evaporation, or under vacuum conditions [139].

Release of drug molecules from this type of controlled release drug delivery system is controlled at a pre-programmed rate by controlling the:

- Loading dose;
- Polymer solubility of the drug, and;
- Diffusivity in the polymer matrix.

An example of this polymer matrix diffusion-controlled drug delivery system is given in the subsection on the Nitro-Dur system.

The drug release rate from the polymer matrix is not constant and can be expressed by the equation

(2) $$\frac{Q}{t^{1/2}={\left(2{\mathrm{AC}}_{R}D_{p}\right)}^{1/2}},$$

where, Q/t^1/2^ is the rate of release of the drug, A is the initial drug loading dose in the polymer matrix, C_R_ is the drug solubility in the polymer and D_p_ is the diffusivity of the drug in the polymer matrix.

#### 5.2.6. Nitro-Dur System

**Figure 17:**
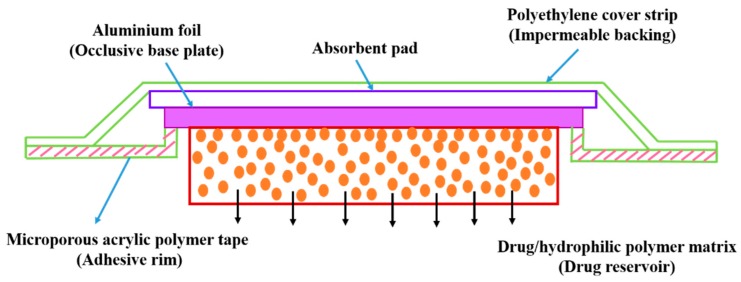
Nitro-Dur system (adapted from [140]).

This type of system is fabricated by heating a hydrophilic polymer, glycerol, and polyvinyl alcohol and then lowering their temperature to develop a polymer gel. Nitrogylcerin and lactose are mixed and dispersed in the polymer, which is solidified at room temperature to form a medicated disc by molding technique. This is assembled into a metallic plastic laminate where the transdermal patch is developed with an adhesive rim. This patch is designed for application to the skin for continuous release of 0.5 mg/cm^2^ for angina treatment. Poly (isobutylene) or poly (acrylate) adhesive are the polymers used in this method and this polymeric adhesive is spread by solvent casting technique over the area of the drug impermeable membrane (Figure 17) [140]. The marketed formulations are Frandol tape by Toaeiyo/Yamanouchi in Japan—isosorbide dinitrate [141]; Nitro-Dur II by Key in the United States—nitroglycerin, which replaces Nitro-Dur, a first generation patch from the market;Habitrol and Nicotrol—nicotine (smoking cessation) [142]; Minitran—nitroglycerin [143], Testoderm—testosterone (testosterone replacement therapy) [144], and Climara—estradiol (vasomotor system treatment associated with menopause) [145].

#### 5.2.7. Compudose

**Figure 18:**
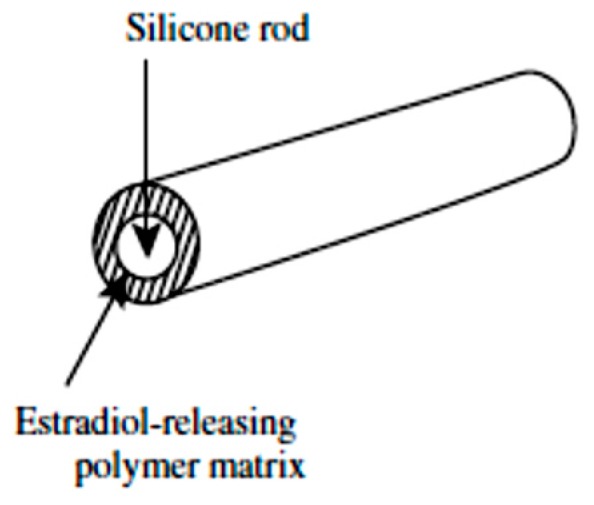
Compudose implant.

This is a subdermal implant formulated on the basis of dispersing estradiol crystals in a viscous silicone elastomer and coating the dispersion over a silicone rod by extrusion technique to form a cylindrically-shape implant (Figure 18). Compudose is subcutaneously implanted for a duration of 200 to 400 days for the delivery of estradiol [146,147]. 

#### 5.2.8. Polymeric Membrane-Based Drug Delivery Systems as Commercialized Formulations 

Polymers form the basis of a significant number of membrane-based drug delivery systems. In fact, advances in the field of polymer sciences have paved the way for a large number of membrane-based drug delivery system designs that have considerable flexibility and functions [148,149,150]. Table 2 summarizes various formulations of drug delivery systems emphasizing the physico-chemical and mechanical properties of various polymers being used in commercially available membrane drug delivery systems.

### 5.3. Future Challenges

The next generation of inorganic particulates and membrane materials could be intended for flow regulation, screening of size, and dynamic pore sizing by means of external controls. Another possibility for fabricating these inorganic particulates and membranes is by the use ofa lab on chip microfluidic model to be used in medical diagnostics. Surface modification of inorganic particulates and membranes with organic and inorganic materials is now being explored. Moreover, when the surface of the inorganic particulates and membranes is modified with grafted polymers, in vitro testing shows promising results. Surface modification using polymers which undergoes conformational changes with a response to stimuli such as temperature, pH, and concentration of ions has shown acceptable results as well. The in vivo tests are necessary to confirm effective delivery and biocompatibility. Many challenges are there in manufacturing biocompatible inorganic particulates and membranes for in-vivo drug delivery applications. The key challenge is to fabricate inorganic particulates and membranes which respond to various stimuli. These inorganic particulates and membrane systems could therefore be used in micro/nanoscale chips for rate-controlled programmable delivery of drugs. 

## 6. Conclusions

Inorganic nanomaterials have emerged as promising materials in different fields, including drug delivery, hard tissue regeneration and bio-imaging. Their exceptional properties, such as tunable stability, functionality, high surface area, and low inherent toxicity, make them the materials of choice for efficient use in targeting. The development of NPs for the enhancement of suitable biomedical applications and decreasing the permeability of relapse is still to be achieved. Membrane materials are essential for biomedical applications such as drug delivery, targeted drug delivery, and other medical applications. The main properties of these membranes include having an average pore size of about a few µm to nm or less, with a narrow size distribution and even smaller thickness. In addition, the membranes also possess high flux, adequate mechanical strength, and chemical stability. It is also obvious that an interdisciplinary approach is important for developing membranes for diverse biomedical applications. As discussed in this article, advanced fabrication techniques are necessary to develop well-ordered, monodisperse, and ultrathin membranes. The market for inorganic NPs and membrane systems is expected to grow steadily in the future.

## Figures and Tables

**Table 1:** Inorganic nanocarriers for drug delivery.

Inorganic Carrier	Drug Loaded	Purpose	References
CaP	Docetaxel	Breast, lung, and ovarian cancer.	[8]
CaP, dopamine nanographene oxide (NGO)	Methotrexate	Anti-rheumatic drug, breast adenocarcinoma.	[38]
Heparin/CaCo/CaP	Doxorubicin hydrochloride	Breast, lung, bladder, stomach, and ovarian cancer, and leukaemia.	[18]
Porous silica CaP	5-fluorouracil	Mammary tumors	[8,20]
NGO	SN38	Colon cancer	[31,32,33,34,35,36]
Carboxylate NDs	Purvalanol and 4-hydroxytamoxitan	Liver and breast cancer	[40,41,42]
Single-walled carbon nanotubes (SWNTs)-polyethylene glycol (PEG), NGO-PEG and MSNPs-GO-chitosan (CHI)	Doxorubicin	Leukaemia, breast cancer, gastric cancer, head and neck cancer, Hodgkin’s lymphoma, liver cancer, kidney cancer, ovarian cancer, small cell lung cancer, soft tissue sarcoma, thyroid cancer, bladder cancer, uterine sarcoma.	[53,54,55]
NDs-NaCl		HT-29 colorectal cancer cells.	[43]
Gold NPs	Paclitaxel	Breast, lung, and pancreatic cancer.	[49]

**Table 2:** Various formulations of drug delivery systems emphasizing the physico-chemical and mechanical properties of various polymers being used in commercially available membrane drug delivery systems.

Polymers	Structure	Fabrication Method	Commercial Products/Literatures	Comments	Reference
Polycarbonate (PC)	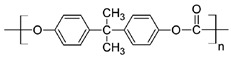	Ion-track etching	Estrogen	Excellent stability against oxidation and biodegradation and improves antifouling properties	[151]
Polyethylene (PE)	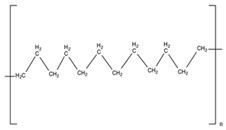	Ion-track etching	Catapress (Clonidine), Boehringer IngelheimClimara (Estradiol), Berlex	Physico-chemical stability andordered pore formation with superior membrane performance	[152]
Polyethylene terephthalate (PET)	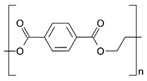	Lithography	Ketoprofen	Biostable, antifouling, has better performance of membranes, in useful in preparing surgical meshes and ligaments	[153]
Polystyrene (PS)	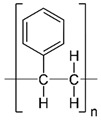	Lithography	d-limonene, ibuprofen	Chemical resistance, easy processing, lower cost, exhibits enhancements in strength, stiffness, toughness, and ductility	[154]
PC, PE	-	Ion-track etching	Estraderm (Nitroglycerin), Rotta Research	Cost-effective and biocompatibility is fairly good	[155]
PC, PE, PET, PS	-	Phase separation	Deponit (Nitroglycerin), Pharma SchwarzHabitrol (Nicotine), Novartis	Cost-effective and biocompatibility is fairly good	[154,156]
Polyurethane (PU)	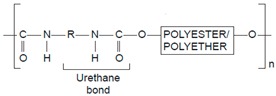	Sol-gel/solvent casting	Vivelle (Estradiol), Novartis	Good elasticity, biodegradable, suitable for hydrophilic drugs, biocompatibility is fairly good	[154]
Polysiloxane (silicone)	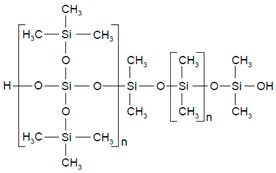	Sol-gel/solvent casting	Prostep (Nicotine), Lederle, Transderm Nitro (Nitroglycerin), AlzaSyncro-Mate-C (Norgestomet)	Better insulation, excellent biocompatibility, and fabricated easily for hydrophilic drugs	[157]
Polyisobutylene (PIB)	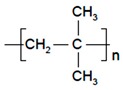	Solvent casting	Aminopyrene, Mitsubishi Petrochem Co., Japan	Good adhesive drug impermeable layer and high degree of tack or self-adhesion	[158]
Polymethyl methacrylate (PMMA), poly (2-hydroxy ethyl methacrylate)	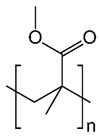	Layer by layer deposition	Androderm (Testosterone), SmithKline Beecham	Physical strength and transparency	[159]
Polyvinyl alcohol (PVA), Poly (ethylene-co-vinyl acetate)	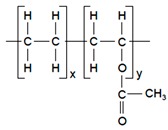	Solvent casting	Nitro-Dur I (Nitroglycerin), Key PharmaTestoderm TTS (Testosterone), Alza	Rate-controlling membranes, high membrane permeability, hydrophilicity and strength, suitable for lipophilic drugs	[160]
Polyacrylic acid, polyacrylate, polyacrylamide	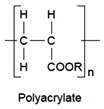	Layer by layer deposition	Epinitril (Nitroglycerin), Rotta ResearchMonsanto (Fentanyl), Dow Corning	Good adhesivity and spreadability and contains a drug impermeable layer	[160]
Polylactides (PLA), polylactic-co-glycolic acid (PLGA), polyglycolides (PGA)	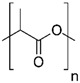 , 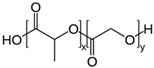	Sol-gel/solvent casting	Propranalol, Exxon Chemical Co.	Good biocompatibility; lactic and glycolic acids are the degradation products and they are easily eliminated from the body	[161,162]
Polyvinyl pyrrolidone (PVP), poly (N-vinyl pyrrolidone)	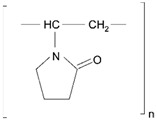	Sol-gel/solvent casting	Cytarabine, ara-ADA, Polyscience	Superior biocompatibility, has suspension capabilities, antinucleating agent, and enhances release rate	[163]
Polyethylene glycol (PEG)		Sol-gel/solvent casting	Miconozale, Rohm, Germany	Chemically inert and free of leachable impurities	[164]
Oxide plus polymer	-	Sol-gel/solvent casting		Superior biocompatibility and has narrow pore size	
Polymer coating on support membrane	-	Layer by layer deposition			[165]
